# Curcumin and Heme Oxygenase: Neuroprotection and Beyond

**DOI:** 10.3390/ijms20102419

**Published:** 2019-05-16

**Authors:** Emanuela Mhillaj, Andrea Tarozzi, Letizia Pruccoli, Vincenzo Cuomo, Luigia Trabace, Cesare Mancuso

**Affiliations:** 1 Fondazione Policlinico Universitario A. Gemelli IRCCS, 00168 Rome, Italy; mhillaj.emanuela@gmail.com; 2 Università Cattolica del Sacro Cuore, 00168 Rome, Italy; 3 Department for Life Quality Studies, Alma Mater Studiorum, University of Bologna, 47900 Rimini, Italy; andrea.tarozzi@unibo.it (A.T.); letizia.pruccoli2@unibo.it (L.P.); 4 Department of Physiology and Pharmacology “V. Erspamer”, Sapienza University of Rome, 00185 Rome, Italy; vincenzo.cuomo@uniroma1.it; 5 Department of Clinical and Experimental Medicine, University of Foggia, 71122 Foggia, Italy; luigia.trabace@unifg.it

**Keywords:** curcumin, free radicals, heme oxygenase, neuroprotection, safety profile

## Abstract

Curcumin is a natural polyphenol component of *Curcuma longa Linn*, which is currently considered one of the most effective nutritional antioxidants for counteracting free radical-related diseases. Several experimental data have highlighted the pleiotropic neuroprotective effects of curcumin, due to its activity in multiple antioxidant and anti-inflammatory pathways involved in neurodegeneration. Although its poor systemic bioavailability after oral administration and low plasma concentrations represent restrictive factors for curcumin therapeutic efficacy, innovative delivery formulations have been developed in order to overwhelm these limitations. This review provides a summary of the main findings involving the heme oxygenase/biliverdin reductase system as a valid target in mediating the potential neuroprotective properties of curcumin. Furthermore, pharmacokinetic properties and concerns about curcumin’s safety profile have been addressed.

## 1. Introduction

Curcumin (1,7-bis[4-hydroxy 3-methoxy phenyl]-1,6-heptadiene-3,5-dione) is a polyphenol compound contained in the rhizome of *Curcuma longa Linn*. Indeed, turmeric contains several polyphenols, the most abundant being curcumin (~77%), demethoxycurcumin (~15%), and *bis*-demethoxycurcumin (~3%) [1]. Considering that curcumin prevails over the other congeners, most of the literature in this field has explored the beneficial effects of this compound, although a few papers have studied the physical and biological properties of related curcuminoids [2,3].

**Figure 1:**
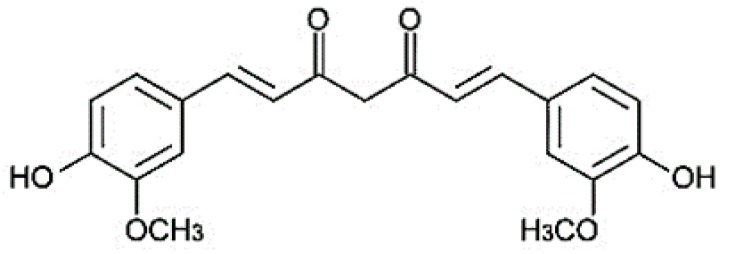
Chemical structure of curcumin.

In addition to the culinary use due to its spicy and pleasant taste, curcumin has been considered for thousands of years, by traditional Indian medicine, as an effective remedy in the treatment of several diseases [4,5,6]. Chemically speaking, the curcumin structure presents two aromatic rings holding *o*-methoxy phenolic groups, linked by an α,β-unsaturated β-diketone moiety (Figure 1) [7].

These three reactive functional sites are responsible for the multiple different biological effects of curcumin. Indeed, literature data have reported that the antioxidant activity of curcumin as a free radical scavenger is mediated primarily by the phenolic groups, which undergo oxidation through electron transfer and hydrogen abstraction mechanisms (reviewed in [8]). On the other hand, many studies have demonstrated that curcumin exerts beneficial effects by enhancing the cell stress response in several experimental models, thus supporting the adjuvant role proposed for this dietary supplement in free radical-derived disorders, mainly neurodegenerative diseases [6,9]. In this light, several research studies underlined the pivotal role played by the heme oxygenase/biliverdin reductase system (HO/BVR) as a determinant of curcumin’s neuroprotective effects (see below). Unfortunately, despite the huge amount of preclinical studies confirming the pleiotropic effects of curcumin due to HO modulation, the clinical evidence is not strong enough to include chronic curcumin supplementation as an effective strategy to prevent or contrast neurodegeneration. One of the reasons behind the dichotomy between preclinical and clinical results has been identified in curcumin pharmacokinetics in humans; first of all, the poor bioavailability after ingestion and the effective concentrations reached in tissues. However, several efforts have been made over recent years to overcome these limitations, with encouraging results.

The aim of this review is to summarize the preclinical and clinical outcomes which have appeared in the scientific literature, supporting or contrasting the claimed therapeutic efficacy of curcumin in neurodegeneration. The reason why the focus has been on the HO/BVR system depends on the several lines of evidence highlighting its role as a determinant of curcumin neuroprotection. Finally, some safety issues related to curcumin supplementation have been also reported.

## 2. The Heme Oxygenase/Biliverdin Reductase Pathway

Heme oxygenase catalyzes the oxygen- and NADPH-dependent oxidation of hemoproteins’ heme moieties at the alpha-meso carbon bridge, yielding equimolar amounts of ferrous iron, carbon monoxide (CO), and biliverdin (BV), the latter being further reduced into bilirubin (BR) by biliverdin reductase [10,11]. Heme oxygenase exists as two main isoforms, named HO-1 and HO-2. Although these isozymes share the same mechanism of action, their regulation and distribution are quite different. Heme oxygenase-1 is the inducible isoform and both its gene transcription and protein levels increase in response to free radicals, e.g., reactive oxygen species and reactive nitrogen species (ROS and RNS, respectively) [11]. Furthermore, HO-1 is the major isoform detected in both the liver and spleen, even if it is expressed, at lower levels, in some brain areas, such as the hippocampus and hypothalamus [11,12]. Conversely, the constitutive isoform HO-2 is involved in the physiological turnover of heme and is mainly detectable in neurons and testes [13,14].

The cytoprotective effects of the HO/BVR system depend on several factors: (i) the degradation of heme, which may become toxic under unbalanced redox conditions; (ii) the generation of CO, which improves mitochondrial biogenesis, counteracts NADPH oxidase-induced ROS generation, activates pro-survival systems (e.g., the protein kinase B/Akt and extracellular signal-related kinase (ERK)/p38 mitogen-activated protein kinase (MAPK) signaling pathways), modulates the release of neuroinflammatory mediators (e.g., interleukin-1β and prostaglandins), dilates cerebral and peripheral vessels, and inhibits platelet aggregation; (iii) the antioxidant and antiviral activities of BR [14,15,16,17,18,19,20]. Interestingly, the modulation of both mitochondrial respiratory chains and NADPH oxidase accounts for CO’s antiproliferative effects [21].

Under oxidative stress and inflammatory conditions, several transcription factors, including nuclear factor erythroid 2-related factor 2 (Nrf2), nuclear factor k-light-chain-enhancer of activated B cells (NF-kB), and hypoxia-inducible factor 1 (HIF1), are established as pivotal regulators of HO-1 induction in the brain [22,23]. Among these transcription factors, Nrf2 plays the conservative role of a positive regulator of HO-1 induction in the development and progression of many diseases [24]. Conversely, a few negative regulators, such as Keap-1 and Bach1, can modulate the crosstalk between the Nrf2 and HO-1 [25,26].

## 3. Curcumin, Neuroprotection, and the HO/BVR Pathway

Over the last 15 years, many papers have appeared in the scientific literature dealing with the cytoprotective effects of curcumin through the up-regulation of HO-1 (see Table 1).

The following are the main studies supporting the neuroprotective effects of curcumin via the modulation of the HO/BVR pathway.

Scapagnini et al. [60] have shown how curcumin (5–25 µM) up-regulates HO-1 in cultured rat hippocampal neurons and, thus, the polyphenol enhances the cell stress response against glucose oxidase-mediated oxidative damage. Shin et al. [61] reported that curcumin (200 mg/kg by intraperitoneal route (i.p.)) reduced kainic acid-induced seizures in mice through the increased expression of HO-1 and endothelial nitric oxide synthase (eNOS) in hippocampal astrocytes, whereas Park and Chun [62] demonstrated that curcumin (0.1–10 µM) reduces oxidative stress, apoptosis, and mitochondrial damage through the direct involvement of HO-1 in BV-2 microglial cells.

These early studies were followed by several others describing the neuroprotective effects of curcumin in neurovascular disorders. Curcumin (100 mg/kg i.p. or 5–30 µM), via HO-1 over-expression, was neuroprotective in a rat model of focal ischemia [63] and in rat cerebellar granule neurons exposed to hemin [64]. In an experimental system of rat hypoxic-ischemic brain injury, curcumin (150 mg/kg per os for three days) overexpressed HO-1 with a mechanism related to Nrf2 nuclear translocation [65]. In addition, curcumin (1–100 µM) has been shown to up-regulate HO-1 and, through this mechanism, it prevents oxygen glucose deprivation-induced damage in rat brain microvascular endothelial cells, a model mimicking the blood–brain barrier (BBB) function [66].

With regard to neurodegenerative diseases, in a rodent model of Alzheimer’s disease (AD), e.g., the SAMP8 mouse, 500 mg/kg of curcumin in a five month diet increased *HO-1* gene expression, together with regulators of mitochondrial function, e.g., the translocator protein (TSPO) [67]. Similarly, by up-regulating HO-1, curcumin (1.25–20 µM) inhibited programmed cell death and prevented the loss of mitochondrial function in SH-SY5Y neuroblastoma cells transfected with appoptosin, a pro-apoptotic protein overexpressed in AD [68]. Concerning neurodegenerative diseases, curcumin (100 mg/kg twice a day for 50 days intragastrically) contrasted extrapyramidal symptoms and increased HO-1 expression, through Akt/Nrf2 phosphorylation, in the substantia nigra pars compacta of rats treated with rotenone, a pharmacological tool able to destroy dopaminergic neurons and, therefore, used to induce experimental Parkinson’s disease (PD) [69]. It is no longer a hypothesis that the cytoprotective effects of curcumin against neuroinflammation depend on the inhibition, HO-1-mediated, of cytokine release and iNOS overexpression in rat microglia [70,71].

Finally, curcumin (15 µM or 200 mg/kg for four days) has been shown to counteract both hydrogen peroxide-induced damage in human retinal pigment cells [72] and cisplatin-induced ototoxicity in outer hair cells [73].

As far as the modulation of HO-2 by curcumin and the potential neuroprotective features, only limited evidence is available. As shown by Yin et al. [74], curcumin (5 µM) up-regulated HO-1 but down-regulated HO-2 in *APPswe* transfected SH-SY5Y. In the same experimental system, curcumin was able to activate phosphoinositide 3-kinase (PI3K) and Akt [74]. By keeping this in mind, it is necessary to draw the conclusion that in selected experimental settings, the neuroprotective outcomes of curcumin strictly depend on the fine-tuning of the HO-1/HO-2 balance, in concert with the modulation of other pro-survival systems, such as PI3K and Akt.

An accurate analysis of both previous paragraphs and Table 1 has drawn attention to the fact that the concentrations of curcumin responsible for protective effects on various organs and tissues, primarily on the brain, were obtained with polyphenol concentrations in the micromolar size range. That said, curcumin, per os, has about a 60% bioavailability, due to a marked first-pass metabolism [9,75]. This implies a low concentration of curcumin in both blood and tissues, even at high doses. Curcumin plasma levels up to 0.16 µM have been detected in humans treated with polyphenol at supra-maximal doses (10–12 g/day), whereas at the lowest doses, curcumin (450–3600 mg/day for one week) reached the plasma concentration of about 0.003 µM [76,77]. In chronic administrations, curcumin (1–4 g/day for six months) exhibited plasma concentrations in the range of 0.06–0.27 µM [78]. With regard to tissue levels, the available data are quite limited. In patients suffering from colorectal cancer and treated with curcumin (1.8 to 3.6 g/day for seven days), concentrations of polyphenol in colorectal tumor tissue and normal tissue were about 7 nmol/g and 20 nmol/g, respectively [79]. These data lead to the conclusion that the plasma concentrations of curcumin that can be reached in the plasma, even after high dose chronic supplementation, are at least two–three orders of magnitude lower than those at which the polyphenol has shown therapeutic effects in in vitro preclinical models. The calculation of the concentrations of curcumin in the tissues is more difficult and may appear less accurate. In the brain, which is protected by BBB, the achievable curcumin concentrations are even lower than those detected in the blood and other tissues. These analytical data have important consequences also from a functional point of view. In subjects with AD, supplementation with curcumin (1–4 g/day for six months) reduced neither peripheral biomarkers of inflammation (e.g., isoprostanes) nor amyloid-β-peptide (Aβ) serum levels; importantly, curcumin did not improve cognitive functions—evaluated through the mini-mental status examination test—in AD patients [78]. Concerning the contribution of the HO/BVR system to the cytoprotective effects of curcumin, the study by Klickovic et al. [80] is significant, showing how 10 healthy male subjects treated with 12 g curcumin *per* os, did not have any significant induction of *HO-1* gene and protein in peripheral blood mononuclear cells up to 48 h from treatment.

In order to overcome limitations due to the poor bioavailability after ingestion and the low plasma concentrations, new formulations of curcumin complexed with liposoluble matrices have been developed (for an extensive review on this topic see [81]) (Table 2).

Among the matrices complexed with curcumin, the ones that are better characterized, from a pharmacokinetic viewpoint, are poly(lactic-co-glycolic) acid (PLGA) derivatives, solid lipid nanoparticles (SLN), and N-trimethyl-chitosan (TMC) [82,83]. Preclinical studies in rodents (Table 2) have shown how the complexation of curcumin with these different carriers increases the C_max_ of both SLN and TMC (155 times and 13 times greater than curcumin, respectively) markedly, suggesting a more effective absorption of the active ingredient [82]. Furthermore, the increase in the area under the curve demonstrates how the presence of SLN or TMC can improve curcumin bioavailability by about 135 times and 41 times, respectively [82]. Finally, an approximately 10-fold increase in the half-life (T_1/2_) of curcumin in the case of formulations based on SLN and TMC implies an extension of the time of persistence of the active agent in the body and, therefore, a more prolonged pharmacological action [82]. Unfortunately, no studies are available in the literature on the interaction of such novel curcumin liposoluble formulations and HO. Indeed, few studies which have been carried out using novel gelatin-based water-soluble formulations of curcumin and remarkable results have been reported. The oral administration of water-soluble curcumin (2–10 mg/kg per os for 45 days) increased plasma insulin levels and improved glucose absorption in diabetic rats by up-regulating HO-1 expression in the pancreas and liver [84]. The same authors supported the beneficial effects of water-soluble curcumin (2–10 mg/kg per os up to one week) in an experimental model of erectile dysfunction. At a dose of 10 mg/kg, water-soluble curcumin over-expressed HO-1 and soluble guanylyl cyclase (sGC) as early as 1 h after treatment, with a concomitant increase in intracavernosal pressure. These effects were maintained over one week from treatment [85].

Although not strictly related to any modulation of the HO system, it is worth mentioning a novel formulation of curcumin complexed with exosomes; these latter are extracellular microvesicles (diameter ranging from 30 to 100 nm) able to carry several types of agents, thus enhancing their bioavailability [86]. Interestingly, curcumin-exosome has been shown to improve cognitive function in a preclinical model of AD, through the inhibition of tau hyperphosphorylation via Akt activation [87].

## 4. Curcumin’s Safety Profile

In any case, regardless of whether it is pure curcumin or new liposoluble or water-soluble formulations, it is worth considering the possibility that the administration of high doses of curcumin causes toxic effects. An organic extract, called turmeric oleoresin, containing a high percentage of curcumin (79–85%), at the concentration of 50,000 ppm (equivalent to 2600 mg/kg and 2800 mg/kg in male and female rats, respectively) has been shown to increase the incidence of ulcers, hyperplasia, and inflammation in the forestomach, cecum, and colon of male and female rats supplemented for two years [88]. Increased evidence of small intestine carcinomas in male mice supplemented with curcumin (0.2 mg/kg) has also been described [88]. Furthermore, curcumin (0.5–2% with the diet for either 2 or 12 weeks) exhibited iron-chelating activity in mice, thus suggesting its involvement in the onset of hypochromic anemia [88]. Finally, curcumin (1 g or 4 g per os for one or six months) modestly increased cholesterol plasma levels in Chinese subjects aged 50 years or older [89]. Regarding the interaction with drug-metabolizing enzymes, curcumin has been shown to inhibit not only several subtypes of cytochrome P450 (CYP), such as CYP1A2, CYP2A6, CYP2B6, CYP2C9, CYP2D6, and CYP3A4, but also uridine dinucleotide phosphate glucuronosyltransterases (UGT), sulfotransferase, glutathione-S-transferase, and organic anion transporting polypeptides (OATP) [9,75,90]. Among the drugs metabolized by these enzymes, whose blood levels may be altered by curcumin and for which further research is needed to assess the effects in cases of chronic supplementation, there are midazolam, talinolol, nifedipine, rosuvastatin, docetaxel, warfarin, clopidogrel, and norfloxacin ([90] and references therein).

In April 2017, the European Food Scientific Agency (EFSA) pointed out that there is no scientific evidence strong enough to justify the use of curcumin in inflammatory diseases, such as osteoarthritis and rheumatoid arthritis [91].

## 5. Conclusions

In this review, we have summarized the conflicting preclinical and clinical results on the neuroprotective effects of curcumin. Furthermore, we have made our best efforts to provide a critical analysis of the pharmacological issues responsible for this divergence, which have precluded the full development of curcumin supplementation as a useful strategy in neurodegenerative diseases. The intriguing results, in terms of improved absorption and bioavailability, obtained with lipid- and water-soluble curcumin formulations, should prompt researchers to transfer this technology to clinical studies, with the hope of overwhelming the pharmacokinetic limitations experienced with standard curcumin. The contribution of pharmaceutical companies to scale up and transpose into clinics these encouraging preclinical results is more than welcome.

## Figures and Tables

**Table 1:** Contribution of HO-1 up-regulation to the biological effects of curcumin in preclinical in vitro and in vivo models.

Preclinical Model	Curcumin (Concentration or Dose)	Effect(s)	Reference(s)
Endothelial cells	2–30 µM	Enhancement of cellular resistance against oxidative damage. Alleviation of vasodilator dysfunction	[27,28,29,30]
Renal tubule cells	1–50 µM	Cytoprotection. Inhibition of fibrosis.	[31,32,33]
Anti-Thy 1 glomerulonephritis rats Nephrectomized rats	100 mg/kg i.p. 75 mg/kg per os	Reduction of renal fibrosis and proteinuria. Inhibition of lipid peroxidation, inflammation and renal fibrosis. Amelioration of renal function.	[34,35]
Hepatocytes	1–50 µM	Cytoprotection against cold/rewarming- or ethanol-induced damages.	[36,37,38]
Monocytes	1–20 µM	Activation of ARE-modulated genes via PKCδ. Inhibition of inflammation.	[39,40]
Macrophages	0.5–50 µM	Inhibition of inflammation.	[41,42,43]
Cardiac myoblasts	5–30 µM	Inhibition of apoptosis. Cytoprotection against cold-storage damage.	[44,45]
Smooth muscle cells	1–20 µM	Inhibition of proliferation.	[46]
LPS-treated mice	30 mg/kg i.p.	Prevention of pulmonary sequestration of neutrophils.	[47]
Pancreatic islets	6–10 µM	Inhibition of islet damage during cryopreservation. Improvement of insulin secretion.	[48,49]
Rat testicular injury	200 mg/kg i.v. 200 mg/kg per os for 30 days before and 45 days after injury.	Inhibition of lipid peroxidation and increase in testicular spermatogenesis. Reduced lipid peroxidation; improvement of serum testosterone level.	[50,51]
Fibroblasts	5–25 µM	Induction of apoptosis and modulation of pathological scar formation.	[52]
High-fat-diet-fed mice	50 mg/kg per os	Improvement in muscular oxidative stress and glucose tolerance.	[53]
Bladder cancer cells	10 µM	Modulation of cancer cell proliferation.	[54]
Breast cancer cells	5–20 µM	Inhibition of tumor invasion.	[55]
Hepatoma cells expressing HCV	5–25 µM	Inhibition of HCV replication.	[56]
Lung cancer cells expressing influenza virus	0.1–10 µM	Inhibition of virus-induced lung injury.	[57]
Keratinocytes	1–30 µM	Anti-inflammatory activity.	[58]
Metabolic syndrome in rats	5 mg/kg i.p. for 6 weeks	Prevention of hyperinsulinemia and amelioration of endothelial-dependent relaxation.	[59]

ARE, antioxidant responsive element; HCV, hepatitis C virus; i.p., intraperitoneal route of administration; i.v., intravenous route of administration; PKC, protein kinase C.

**Table 2:** The main pharmacokinetic parameters of curcumin and some of its novel formulations (adapted from [82]).

Formulation	AUC	C_max_	T_max_	T_1/2_
Curcumin	~312 ng/mL·h ^a^	~ 245 nM ^a^	0.5 h ^a^	~1.0 h ^a^
Curcumin-PLGA	~3224 ng/mL·h ^b^	~ 710 nM ^b^	2.0 h ^b^	
Curcumin-TMC	~12,760 ng/mL·h ^c^	~3.3 μM ^c^	2.0 h ^c^	~12 h ^c^
Curcumin-SLN	~42,000 ng/mL·h ^d^	~38 μM ^d^	0.5 h ^d^	

^a^ Male Sprague-Dawley rats treated with 250 mg/kg curcumin per os; ^b^ male Sprague-Dawley rats treated with 100 mg/kg curcumin-PLGA per os; ^c^ Balb/c mice treated with 50 mg/kg curcumin-TMC per os; ^d^ male Wistar rats treated with 50 mg/kg curcumin-SLN per os; AUC, area under the curve; C_max_, peak plasma concentration; PLGA, poly(lactic-co-glycolic) acid; SLN, solid lipid nanoparticles; T_max_, time necessary to reach the C_max_; T_1/2_, half-life; TMC, N-trimethyl chitosan.
